# The BrainLat project, a multimodal neuroimaging dataset of neurodegeneration from underrepresented backgrounds

**DOI:** 10.1038/s41597-023-02806-8

**Published:** 2023-12-09

**Authors:** Pavel Prado, Vicente Medel, Raul Gonzalez-Gomez, Agustín Sainz-Ballesteros, Victor Vidal, Hernando Santamaría-García, Sebastian Moguilner, Jhony Mejia, Andrea Slachevsky, Maria Isabel Behrens, David Aguillon, Francisco Lopera, Mario A. Parra, Diana Matallana, Marcelo Adrián Maito, Adolfo M. Garcia, Nilton Custodio, Alberto Ávila Funes, Stefanie Piña-Escudero, Agustina Birba, Sol Fittipaldi, Agustina Legaz, Agustín Ibañez

**Affiliations:** 1 Latin American Brain Health Institute (BrainLat), Universidad Adolfo Ibáñez, Santiago, Chile; 2 Escuela de Fonoaudiología, Facultad de Odontología y Ciencias de la Rehabilitación, Universidad San Sebastián, Santiago, Chile; 3 PhD Neuroscience Program, Physiology and Psychiatry Departments, Pontificia Universidad Javeriana, Bogotá, Colombia; 4 Memory and Cognition Center Intellectus, Hospital Universitario San Ignacio, Bogotá, Colombia; 5 Global Brain Health Institute, University of California San Francisco, San Francisco, USA; 6 Global Brain Health Institute, Trinity College Dublin, Dublin, Ireland; 7 Cognitive Neuroscience Center (CNC), Universidad de San Andrés & CONICET, Buenos Aires, Argentina; 8 Department of Neurology, Massachusetts General Hospital and Harvard Medical School, Boston, MA USA; 9 Departamento de Ingeniería Biomédica, Universidad de Los Andes, Bogotá, Colombia; 10 Memory and Aging Clinic, University of California San Francisco, San Francisco, USA; 11 Neuropsychology and Clinical Neuroscience Laboratory (LANNEC), Physiopathology Department - Institute of Biomedical Sciences (ICBM), Neurocience and East Neuroscience Departments, Faculty of Medicine, University of Chile, Santiago de Chile, Chile; 12 Geroscience Center for Brain Health and Metabolism, (GERO), Santiago de Chile, Chile; 13 Memory and Neuropsychiatric Center (CMYN), Memory Unit – Neurology Department, Hospital del Salvador and Faculty of Medicine, University of Chile, Santiago de Chile, Chile; 14 Servicio de Neurología, Departamento de Medicina, Clínica Alemana-Universidad del Desarrollo, Santiago de Chile, Chile; 15 Centro de Investigación Clínica Avanzada (CICA), Facultad de Medicina-Hospital Clínico, Universidad de Chile, Independencia, Santiago, 8380453 Chile; 16 Departamento de Neurología y Neurocirugía, Hospital Clínico Universidad de Chile, Independencia, Santiago, 8380430 Chile; 17 Departamento de Neurociencia, Facultad de Medicina, Universidad de Chile, Independencia, Santiago, 8380453 Chile; 18 Departamento de Neurología y Psiquiatría, Clínica Alemana-Universidad del Desarrollo, Santiago, 8370065 Chile; 19 Grupo de Neurociencias de Antioquia de la Universidad de Antioquia, Medellín, Colombia; 20 School of Psychological Sciences and Health, University of Strathclyde, Glasgow, United Kingdom; 21 Mental Health Department, Hospital Universitario Fundación Santa Fe de Bogotá, Memory Clinic, Bogotá, Colombia; 22 Departamento de Lingüística y Literatura, Facultad de Humanidades, Universidad de Santiago de Chile, Santiago, Chile; 23 Unit Cognitive Impairment and Dementia Prevention, Peruvian Institute of Neurosciences, Lima, Peru; 24 Geriatrics Department, Instituto Nacional de Ciencias Médicas y Nutrición Salvador Zubirán, Mexico City, Mexico; 25 Instituto Universitario de Neurociencia, Universidad de La Laguna, Tenerife, Spain; 26 Facultad de Psicología, Universidad de La Laguna, Tenerife, Spain

**Keywords:** Neurodegeneration, Biomarkers

## Abstract

The Latin American Brain Health Institute (BrainLat) has released a unique multimodal neuroimaging dataset of 780 participants from Latin American. The dataset includes 530 patients with neurodegenerative diseases such as Alzheimer’s disease (AD), behavioral variant frontotemporal dementia (bvFTD), multiple sclerosis (MS), Parkinson’s disease (PD), and 250 healthy controls (HCs). This dataset (62.7 ± 9.5 years, age range 21–89 years) was collected through a multicentric effort across five Latin American countries to address the need for affordable, scalable, and available biomarkers in regions with larger inequities. The BrainLat is the first regional collection of clinical and cognitive assessments, anatomical magnetic resonance imaging (MRI), resting-state functional MRI (fMRI), diffusion-weighted MRI (DWI), and high density resting-state electroencephalography (EEG) in dementia patients. In addition, it includes demographic information about harmonized recruitment and assessment protocols. The dataset is publicly available to encourage further research and development of tools and health applications for neurodegeneration based on multimodal neuroimaging, promoting the assessment of regional variability and inclusion of underrepresented participants in research.

## Background & Summary

Dementia and neurodegenerative diseases significantly impact patients, families, the economy, and public health systems worldwide. However, such impact, coupled with prevalence, underdiagnosis, and assessment, is unequal. Latin America is one of the most unequal regions in the world, with a lack of adequate dementia diagnosis and care^1–4^. The current prevalence of dementia in LACs is estimated at 8.5% and is projected to be 19.33% by 2050, representing an increase of 220% approximately. Such prevalence is higher compared to other regions^5,6^ including Europe (current 6.9% and projected up to 7.7% by 2050) or North America (current 6.5% and projected up to 12.1% by 2050)^4,5,7–10^ Paradoxically, most global research on neurodegeneration is underrepresented in terms of Latino populations^4,8,11–14^ Most literature arises predominantly from the US, Europe, and other regions with high-income settings. Despite the pressing need to evaluate regional diversity and provide tailored evidence for underrepresented samples^2,15–18^, current scientific findings on neurodegeneration in Latin America do not meet this requirement. The situation seems more urgent given the recent evidence that the so-called non-stereotypic populations^15^ (participants from underrepresented populations in admixtures, genetics, cultural backgrounds, and demographics) defy the generalization of brain-phenotype models from stereotypical populations^19–22^. Thus, to evaluate diversity in dementia research is an immediate and significant gap that needs to be addressed.

Developing affordable, scalable, and widely available biomarkers is crucial for early diagnosis and intervention, specially Latin America^4,8,11–14^. While several multimodal neuroimaging databases and consortia for neurodegeneration exist (e.g., ADNI, LONI, HCP, UK Biobank, CAMCAN, ABCD, PPMI, ENIGMA), there is a lack of datasets from underrepresented, non-stereotypical samples, and few databases include EEG data. EEG is an advantageous technique for assessing neurodegeneration due to its cost-effectiveness, accessibility, scalability, and applicability to underserved populations. The opportunity to evaluate brain dynamics and networks with combined spatiotemporal methods represents a significant advance for clinical assessment^23,24^, as well as multimodal imaging and computational approaches to neuroscience^25–27^. However, to our knowledge, no other open datasets of multiple neurodegenerative diseases include resting-state recordings with high spatial (fMRI) and temporal (EEG) resolution.

**Fig. 1:**
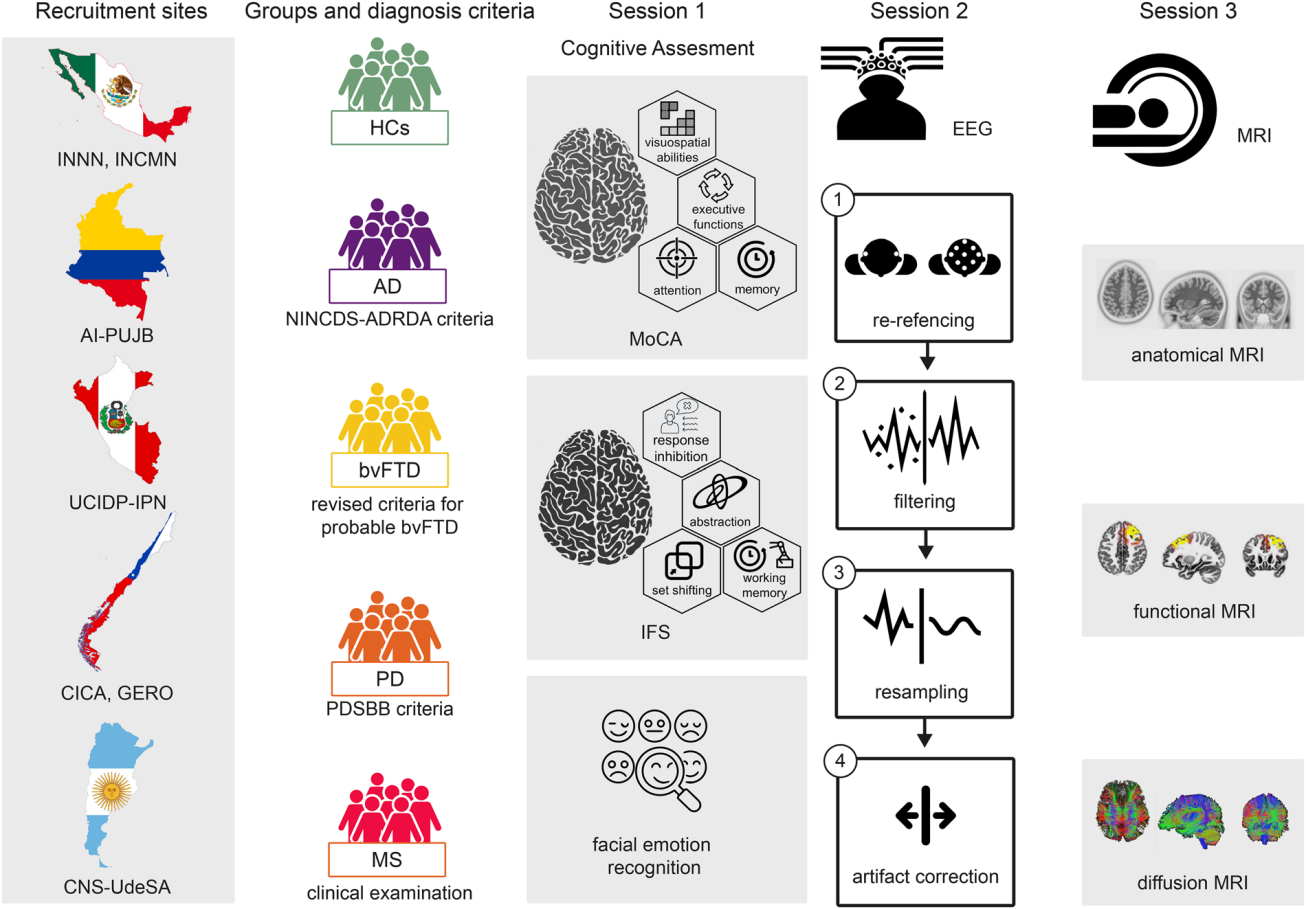
The BrainLat multimodal dataset of neurodegenerative diseases. The figure summarizes the entire protocol, encompassing various centers, participant groups, diagnostic criteria, cognitive assessments, and EEG and MRI recordings. The activities carried out by the participants during their three visits to the clinical center are also depicted. For the EEG session, the figure illustrates the key steps in the processing pipeline. Session three summarizes the different MRI recordings (anatomical, functional, and diffusion MRI). The recruitment sites included the INNN: Instituto Nacional de Neurología y neurocirugía, Ciudad de México, Mexico; INCMN: Geriatrics Department, Instituto Nacional de Ciencias médicas y nutrición Salvador Zubirán, Mexico City, Mexico; AI-PUJB: Aging Institute, Pontificia Universidad Javeriana, Bogotá, Colombia; UCIDP-IPN: Unit Cognitive Impairment and Dementia Prevention, Peruvian Institute of Neurosciences, Lima, Peru; CICA: Centro de Investigación Clínica Avanzada (CICA) Hospital Clínico Universidad de Chile, Chile: GERO: Neurology Department, Geroscience Center for Brain Health and Metabolism, Santiago, Chile; CNC-UdeSA Centro de Neurociencia Cognitiva, Universidad de San Andrés, Argentina. AD: Alzheimer’s disease, bvFTD: behavioral variant frontotemporal dementia, PD: Parkinson’s disease, MS: Multiple sclerosis, HCs: older healthy controls.

The BrainLat dataset^28^ (Fig. 1) is a pioneering dataset that addresses these gaps by providing data from a diverse group of Latin American patients with various neurodegenerative diseases, including Alzheimer’s disease (AD), behavioral variant frontotemporal dementia (bvFTD), Parkinson’s disease (PD), multiple sclerosis (MS), and healthy controls. It is a regional effort designed as a multicentric study with harmonized recruitment and neurocognitive assessment, led by the Latin American Brain Health Institute (BrainLat)^29^ and the Multi-partner consortium to explore dementia research in Latin America (ReDLat)^10,30,31^ with the support of various stakeholders. Details for harmonizing per the ReDLat procedures (recruitment and neurocognitive assessment) include a site manual, a checklist, and a tutorial, all available elsewhere^30^.

Along with cognitive and sociodemographic information, the BrainLat dataset^28^ includes anatomical MRI, resting-state fMRI, and resting-state EEG. Neuroimaging records have not been harmonized to allow dataset users to conduct custom analyses. Nevertheless, different post-recording harmonization (w- and z-scores, confusion matrices, data transformation/normalization, optimizers, and k-folds validation) have been successfully applied in this data^32,33^. Thus, the BrainLat dataset^28^ has been utilized for understanding neurodegeneration and developing multimodal markers^32–59^.

By making the BrainLat dataset^28^ openly accessible, the project aims to encourage additional analyses and data exploitation. This dataset^28^ is the first to be released from a larger multicentric initiative, the Euro-LAD EEG consortium^60^, a Global EEG Platform for dementia research inclusive of diverse and underrepresented data. We hope this dataset^28^ will allow the future development of normative EEG datasets based on harmonized multicentric data, assessing sociodemographic variability, and promoting the development of tools and health applications for neurodegeneration based on multimodal neuroimaging.

Latin American populations display extensive heterogeneity triggered by the unique combination of genetic and environmental (i.e., socioeconomic) differences^3,9^. This open-access dataset^28^ fosters collaboration and facilitates the identification of new biomarkers, ultimately contributing to advancements in understanding and treating neurodegenerative diseases. While genetics and socioeconomic status information are not currently included in the BrainLat dataset^28^, we anticipate that these will be available upon completing the ReDLat protocol by 2026, when the dataset will be updated.

## Methods

### Participants

The BrainLat dataset^28^ contains neuroimaging and cognitive data from 780 subjects, including patients with AD (N = 278), bvFTD (N = 163), PD (N = 57) and MS (N = 32), and HCs (N = 250). Participants were enrolled in clinical sites from the Multi-Partner Consortium to Expand Dementia Research in Latin America (ReDLat), a regional effort to harmonize participant enrollment and neurocognitive assessment in multicentric studies^10,30^. Five ReDLat countries were included (Argentina, Chile, Colombia, Mexico, and Peru, see Table 1). The demographic information of the BrainLat dataset^28^ (global information) is presented in Table 2, while the information split for the recruitment sites is presented in Table 3 and stored in BrainLat_Demographic.csv. There was limited information available on the age of the participants at the onset of the disease. Consequently, the duration of the disease is not reported.

**Table 1:** List of sites contributing to the BrainLat dataset.

Short name	Country	Full name	Initials of the PI
INCMN	MX	Geriatrics Department, Instituto Nacional de Ciencias Médicas y Nutrición Salvador Zubirán, Mexico City	AF
CICA	CL	Centro de Investigación Clínica Avanzada (CICA) Hospital Clínico Universidad de Chile	BE
UV	CO	Universidad del Valle, Cali	CA
UCIDP-IPN	PE	Unit Cognitive Impairment and Dementia Prevention, Peruvian Institute of Neurosciences, Lima	CU
CNC-UdeSA	AR	Centro de Neurociencia Cognitiva, Universidad de San Andrés, Buenos Aires	IB
AI-PUJB	CO	Aging Institute, Pontificia Universidad Javeriana, Bogotá	MA
GERO	CL	Neurology Department, Geroscience Center for Brain Health and Metabolism, Santiago	SL
INNN	MX	Instituto Nacional de Neurología y neurocirugía, Ciudad de México	SO

Country codes meet the standards of the International Organization for Standardization (ISO). MX: Mexico, CL, Chile, CO: Colombia, PE: Perú, AR: Argentina. PI: principal investigator.

**Table 2:** Demographic information of the BrainLat dataset.

group	N	demography			
		sex (F: M)	age (years)	education (years)	HbP (right: left)
AD	278	167:111	72.2 (7.9)	11.9 (4.8)	157:5*
bvFTD	163	77:86	65.1 (10.5)	13.1 (5.0)	135:5*
PD	57	19:38	69.9 (11.2)	7.9 (5.5)	55:1*
MS	32	26:6	38.5 (8.9)	16.5 (3.4)	—
HCs	250	164:86	67.9 (8.9)	14.7 (4.3)	177:5*
total	780	453:327	62.7 (9.5)	12.8 (4.6)	524:16*

Age and years of formal education are presented as mean (standard deviation). Sex is the ratio between females (F) and males (M). HbP: Handedness by preference (self-referenced handedness). The symbol *indicates the field contains missing information. The symbol - indicates data is not available. AD: Alzheimer’s disease, bvFTD: behavioral variant frontotemporal dementia, PD: Parkinson’s disease, MS: Multiple sclerosis, HCs: healthy controls.

**Table 3:** Demographic information of the BrainLat dataset split by recruitment site.

group	total	recruitment site		demography				
				N	sex (F: M)	age (years)	education (years)	HbP (right: left)
AD	279	CICA	CL	4	4:00	71.0 (10.1)	5.5 (4.4)	4:0
		UCIDP-IPN	PE	73	30:43	72.5 (8.5)	9.5 (3.6)	71:1*
		CNC-UdeSA	AR	43	31:12	76.0 (6.1)	9.3 (5.8)	32:3*
		AI-PUJB	CO	23	14:90	67.8 (8.7)	11.0 (4.5)	23:0
		GERO	CL	114	65:49	83.0 (7.2)	16.5 (4.7)	29:0*
		INNN	MX	21	10:11	76.8 (6.6)	6.3 (6.9)	18:1*
bvFTD	165	CICA	CL	2	1:1	56.5 (0.7)	14.5 (3.5)	2:0
		UCIDP-IPN	PE	1	0:1	61.0 -	11.0 -	1:0
		CNC-UdeSA	AR	38	17:21	71.5 (17.2)	13.0 (4.2)	27:1*
		AI-PUJB	CO	81	46:35	61.0 (7.7)	13.3 (5.1)	78:3
		GERO	CL	35	10:25	74.3 (10.5)	13.8 (5.1)	23:0*
		INNN	MX	6	4:2	68.8 (7.0)	8.5 (6.2)	4:1*
PD	57	CNC-UdeSA	AR	11	5:6	74.0 (6.4)	10.8 (5.6)	11:0
		UV	CO	25	3:22	64.5 (7.4)	11.1 (5.8)	28:0
		GERO	CL	21	11:10	73.8 (6.9)	10.9 (5.4)	19:1*
MS	32	CNC-UdeSA	AR	32	26:6	38.5 (8.9)	16.5 (3.4)	—
HCs	250	INCMN	MX	2	2:0	65.0 (1.4)	15.0 -	*
		CICA	CL	28	18:00	63.8 (5.2)	12.7 (4.7)	28:0
		UCIDP-IPN	PE	7	3:7	77.4 (9.0)	12.0 (1.9)	7:0
		CNC-UdeSA	AR	80	58:22	66.4 (9.0)	17.1 (3.3)	65:4*
		AI-PUJB	CO	46	30:16	60.2 (8.6)	14.4 (4.7)	45:1
		GERO	CL	87	60:17	73.3 (5.2)	13.7 (4.0)	30:0*

Age and years of formal education are presented as mean (standard deviation). Country codes meet the standards of the International Organization for Standardization (ISO). Sex is the ratio between females (F) and males (M). HbP: Handedness by preference (self-referenced handedness). The symbol * indicates the field contains missing information. The symbol - indicates data is not available. AD: Alzheimer’s disease, bvFTD: behavioral variant frontotemporal dementia, PD: Parkinson’s disease, MS: Multiple sclerosis, HCs: healthy controls, AR: Argentina, CL, Chile, CO: Colombia, CNC- UniSA: Centro de Neurociencias Cognitivas, Universidad de San Andrés, Gero-CMYN: Clínica de Memoria y Neuropsiquistría, Centro de Gerociencia, Salud Mental y Metabolismo.

As noted above, the BrainLat dataset^28^ included MS patients, where primary mechanisms are considered to have a larger inflammatory component compared to AD, bvFTD, and PD. Nonetheless, incorporating MS in the dataset holds significant relevance. Comparisons between MS and other neurodegenerative diseases are relevant and frequently reported^47,61,62^. Although the pathophysiological pathways differ, insightful comparisons between these conditions can be made. By leveraging multivariate data, comprehensive analyses can be performed to delineate shared and unique disease patterns^63–66^. Moreover, recent insights have emphasized shared mechanisms across different neurodegenerative diseases, including the role of inflammatory pathways^65–68^. Furthermore, the flexible nature of the dataset design allows for analyses to be conducted with patient groups combined or separated. This offers the opportunity to observe MS alone or in comparison with other conditions, providing a rich perspective in understanding complex neurodegenerative pathways.

### Ethics

The institutional ethic boards of each recruitment site provided ethical approval for collecting and sharing data. The ethics approval reference codes for each participating institution (Table 1) are listed below.

- Geriatrics Department, Instituto Nacional de Ciencias Médicas y Nutrición Salvador Zubirán (INCMN), Mexico City, Mexico (reference code 09-CEI-011-2016-0627).
- Centro de Investigación Clínica Avanzada (CICA) Hospital Clínico Universidad de Chile, Santiago, Chile (reference code FWA00029089).
- Universidad del Valle, Cali, Colombia (reference code FWA00028864).
- Unit Cognitive Impairment and Dementia Prevention, Peruvian Institute of Neurosciences, Lima, Peru (reference code 10360-19).
- Centro de Neurociencia Cognitiva, Universidad de San Andrés, Buenos Aires, Argentina (reference code 0990-0279).
- Aging Institute, Pontificia Universidad Javeriana, Bogotá, Colombia (reference code FM-CIE-0741-19).
- Neurology Department, Geroscience Center for Brain Health and Metabolism, Santiago, Chile (reference code FWA00029089).
- Instituto Nacional de Neurología y neurocirugía, Ciudad de México (reference code 12–20).

The ethics approvals were granted in accordance with the ethical regulations and guidelines of the countries where the centers are located, and in compliance with the Declaration of Helsinki.

On their first visit to the recruitment centers, participants were provided with both oral and written explanations about objectives, risks, and benefits of the study. Afterwards, participants proceed to sign a written consent form (Fig. 1). Patients were accompanied by a relative or legal representative, who signed the informed consent when necessary. The informed consent provided by the participants included for the open publication of the anonymized data. Consequently, participants were educated about processing information to protect the confidentiality of personally identifiable information. Information about sharing and publication of anonymized data was provided. For anonymization, the participants’ names were replaced by a code (section Usage Notes), and MRI images were defaced (section Data Records).

### Recruitment, inclusion criteria, clinical and cognitive assessments

Information about the study was spread through networks of the recruitment centers and social media. The target audience was the HCs, patients with neurodegenerative diseases, and their families. The inclusion and exclusion criteria of the participants are outlined below. These criteria were reviewed and agreed upon by clinicians of the ReDLat consortium^30^.

The inclusion criteria for controls (HCs) were:

- Possessing a Modified Clinical Dementia Rating (CDR) = 0 and a Mini-Mental State Examination (MMSE) score >25.
- Meeting the criteria for fluency in Spanish (judged by the evaluator as sufficient to complete the assessment).
- Having adequate visual and auditory acuity to complete cognitive testing.
- Not having any proven history of substance abuse, or neurological or psychiatric disorders.

The inclusion criteria for participants with neurodegeneration were:

- Having a clinical diagnosis of mild/moderate AD, bvFTD, PD, or MS. When needed, the diagnosis was supported by neuroimaging assessment (routine MRI or hypoperfusion/hypometabolism SPECT or PET).
- Meeting criteria for fluency in Spanish (judged by the evaluator as sufficient to complete the assessment).
- Must have adequate visual and auditory acuity for cognitive testing.
- For patients with dementia (AD and bvFTD): having an informant who maintained frequent contact with the participant (e.g., family member, partner, friend, caregiver). The informant should be familiar with the participant’s daily activities and able to provide information on the participant’s cognitive and functional status. The duration of acquaintance with the patient should be at least six months.
- Being able to sign the informed consent or be accompanied by an authorized representative who could do so.

The exclusion criteria for participants with neurodegenerative diseases were:

- Mini-Mental State Examination (MMSE) score <14 (all groups), CDR = 3 (for AD), or FTLD-CDR (FTD) = 3.
- Intoxication at the time of evaluation; multiple system atrophy, brain tumor, prion disease, Huntington’s disease, intracerebral hemorrhage, stroke.
- Presence of ferromagnetic implants impacting MRI acquisition.
- Clinically significant ischemic or hemorrhagic cerebrovascular disease, diffuse confluent white matter lesions (Fazekas Grade 3), intra or extra-axial masses revealed by MRI that compress brain parenchyma and that may affect cognition and/or behavior or may confound imaging analysis.
- Deficiency of B12 (B12 < normal), hypothyroidism (TSH >150% of normal), HIV infection, renal insufficiency (creatinine >2), liver insufficiency (AST >2x normal), respiratory insufficiency (requiring oxygen), other significant systemic diseases (as judged by the attending neurologist).
- Basic clinical criteria for other types of dementia or other neurological disorders.
- Inability to communicate in Spanish.

Patients fulfilled either the current criteria of the National Institute of Neurological Disorders and Stroke–Alzheimer Disease and Related Disorders (NINCDS-ADRDA) working group for probable AD^69^, the revised criteria for probable bvFTD^70^, or the criteria of the United Kingdom Parkinson’s Disease Society Brain Bank (PDSBB) for PD^71^. Patients with MS were diagnosed by experts, considering standard clinical examination, magnetic resonance imaging, and lumbar puncture when necessary^40^.

Patients with AD and bvFTD were functionally impaired, as verified by caregivers. The AD patients were all sporadic, except for those recruited by one of the Colombian sites, who had PSEN1 mutations. The PD and AD groups had typical disease presentations, apart from the AD patients with PSEN1mutations that exhibited early-onset symptoms. The BraiLat dataset^28^ does not include records of late-onset AD or other atypical disease presentations. Additionally, participants with bvFTD exhibited noticeable changes in personality and social behavior. Participants with PD received levodopa treatment and were evaluated during the ‘on’ phase. Further details regarding this medication are unavailable.

ñA comprehensive assessment of the neurological, neuropsychiatric, and neuropsychological domains of the participants was conducted by ReDLat experts using semi-structured interviews and standardized cognitive and functional tests. The evaluation lasted up to three hours and comprised the test described below. The cognitive outcomes are stored in BrainLat_Cognition.csv.

### Clinical assessments

#### Clinical dementia rating scale (CDR)

The CRD is an 8-item dementia rating scale that assesses cognitive and functional decline. Scores: 0 = Healthy, 0.5 = questionable dementia, 1 = mild dementia, 2 = moderate dementia, 3 = severe dementia^72,73^. Only AD patients were evaluated with this instrument.

#### Frontotemporal lobar degeneration-modified clinical dementia rating (FTDL-CDR)

The FTDL-CDR is a 5-point scale characterizing six cognitive and functional domains: memory, orientation, judgment and problem solving, community affairs, home and hobbies, and personal care^73^. Additionally, it is used for assessing behavioral and motor domains in the case of the frontotemporal dementia spectrum. Only bvFTD patients were evaluated with this instrument.

#### Section 3 of the movement disorder society-sponsored revision of the unified parkinson’s disease rating scale (MDS-UPDRS-III)

The MDS-UPDRS-III^74^ is a revised and expanded version of UPDRS^75^ consisting of twenty questions that needed to be answered by the patient or caregiver. The MDS-UPDRS has four parts, with part III dedicated to motor complications. The stage of the disease was rated with the Hoehn & Yahr (H&Y) scale^76^. Only PD patients were evaluated with these instruments.

#### The multiple sclerosis severity score (MSSS)

The MSSS^77^ relates scores of the Expanded Disability Status Scale (EDSS)^78^ to the distribution of disability in patients with comparable disease durations for detecting rates of disease progression. Only MS patients were evaluated with this instrument.

### Cognitive tools

#### The montreal cognitive assessment (MoCA)

The MoCA^79^ is a cognitive screening for tracking mild cognitive impairment. The MoCa comprises 30 points evaluating short-term memory, visuospatial abilities, multiple aspects of executive functions, attention, memory, and working memory, language abilities, and orientation to time and place. Its maximum score is 30, with higher scores indicating better performance. All participants were evaluated with this tool.

#### The ineco frontal screening (IFS)

The IFS^80^ is a tool for screening executive function in patients with neurodegenerative diseases. The IFS evaluates response inhibition and set shifting, the capacity of abstraction, and working memory. The maximum score on the test is 30, with higher scores indicating better performance. All participants were evaluated with this tool.

#### Facial emotion recognition (FER)

In this task, participants identify emotional expressions depicted in a series of photos^39^ (thirty-five faces selected from the emotion face set^81^). Participants are instructed to associate faces with one of six possible emotions (happiness, surprise, sadness, fear, disgust, anger) or a neutral expression. A score (max. 15) is calculated from the percentage of correct responses. All HCs, AD, bvFTD, and PD participants were evaluated with this tool.

### Functional ability assessments

#### Functional activities questionnaire (FAQ)

is a 10-item rating scale that measures instrumental activities of daily living (such as preparing meals and personal finance)^82^. A score above 9 suggests a possible impaired function and possible cognitive impairment. All HCs, AD, bvFTD, and PD participants were evaluated with this tool.

#### Frontotemporal dementia rating scale (FRS)

is a 30-item scale that evaluates severity in people with dementia^83,84^. Scores from 1.92 to −2.58 indicate a moderate/severe disease stage and from −2.58 to −6.66 very severe/profound disease stage. Only bvFTD participants were evaluated with this tool.

All clinical, cognitive, and functionality assessments are provided as raw data. However, these can be normalized and harmonized for comparisons as performed elsewhere with the current data^31^.

### Neuroimaging data

EEG and MRI were acquired within 6 months after the neurological evaluation (second and third visits of the participants to each recruitment site), following the ReDLat protocol^10,30^. The duration of the assessment includes up to 2 hours for EEG, and up to 1 hour for MRI.

The duration of the assessment includes up to 2 hours for EEG, and up to 1 hour for MRI. Global information about the neuroimaging modalities and the data split by recruitment sites are presented in Tables 4, 5. Noteworthy, as in other available datasets, ours has some missing data. For most of the participants, one (MRI) or two (MRI + EEG) neuroimaging modalities were acquired (Tables 4, 5). Nevertheless, EEG was the only neuroimaging modality acquired in a reduced group of participants (Tables 4, 5). Reasons for missing data include the different objectives of the studies for which data was initially acquired, technological constraints, and the use of varied data storage formats. Detailed information about the neuroimage modalities acquired from each participant is provided in BrainLat_records.csv, which is deposited on Synapse.

**Table 4:** Global neuroimaging information of the BrainLat dataset.

Group	imagin					EEG
	scan	N	MRI	fMRI	DWI	
AD	1.5	156	155	14	3	35
	3.0	91	90	72	51	
bvFTD	1.5	28	28	15	0	19
	3.0	97	96	82	91	
PD	3.0	55	55	30	30	29
MS	3	31	31	30	0	32
HC	1.5	81	81	27	27	42
	3	177	176	171	161	

Imaging information is presented for anatomical (MRI), functional (fMRI), and diffusion-weighted (DWI) resonance magnetic imaging. The magnetic field of the scan (scan) is presented. N: number of subjects, AD: Alzheimer’s disease, bvFTD: behavioral variant frontotemporal dementia, PD: Parkinson’s disease, MS: Multiple sclerosis, HCs: healthy controls.

**Table 5:** Neuroimaging information of the BrainLat dataset split by recruitment site.

Group	recruitment site		N	imaging			EEG	
				MRI	fMRI	DWI	+MRI	EEG only
AD	CICA	CL	4	4	—	—	—	—
	UCIDP-IPN	PE	66	66	—	—	—	—
	CNC-UdeSA	AR	22	21	21	19	—	—
	GERO	CL	82	81	14	3	—	—
	INNN	MX	4	4	—	—	—	—
	UCIDP-IPN	PE	7	—	—	—	—	—
	CNC-UdeSA	AR	18	18	17	18	13	3
	AI-PUJB	CO	23	22	23	22	—	—
	GERO	CL	26	26	25	11	15	4
	INNN	MX	17	17	7	—	—	—
bvFTD	CICA	CL	1	1	—	—	—	—
	GERO	CL	23	23	15	—	—	—
	INNN	MX	4	4	—	—	—	—
	CICA	CL	1	1	—	—	—	—
	UCIDP-IPN	PE	1	1	—	—	—	—
	CNC-UdeSA	AR	39	39	37	35	12	1
	AI-PUJB	CO	81	80	69	79	—	—
	GERO	CL	13	13	12	11	3	3
PD	UV	CO	25	25	—	—	—	—
	CNC-UdeSA	AR	9	9	9	9	5	2
	GERO	CL	21	21	21	21	20	1
SM	CNC-UdeSA	AR	31	31	30	—	31	1
HCs	INCMN	MX	2	2	—	—	—	—
	UCIDP-IPN	PE	1	1	—	—	—	—
	CNC-UdeSA	AR	3	3	3	—	—	—
	GERO	CL	50	50	0	4	—	—
	CICA	CL	28	28	28	28	—	—
	UCIDP-IPN	PE	6	6	0	0	—	—
	CNC-UdeSA	AR	72	72	72	63	17	1
	AI-PUJB	CO	46	45	46	45	—	—
	GERO	CL	25	25	25	25	13	11

Imaging information is presented for anatomical (MRI), functional (fMRI), and diffusion-weighted (DWI) resonance magnetic imaging. The magnetic field of the scan (scan) is presented. Codes for the recruitment sites are CICA: Centro de Investigación Clínica Avanzada (CICA) Hospital Clínico Universidad de Chile; Santiago, Chile; UCIDP-IPN: Unit Cognitive Impairment and Dementia Prevention, Peruvian Institute of Neurosciences, Lima, Peru; GERO: Neurology Department, Geroscience Center for Brain Health and Metabolism, Santiago, Chile; INNN: Instituto Nacional de Neurología y Neurocirugía, Ciudad de México, Mexico, CNC-UdeSA: Centro de Neurociencia Cognitiva, Universidad de San Andrés, Buenos Aires, Argentina; AI-PUJB: Aging Institute, Pontificia Universidad Javeriana, Bogotá, Colombia; INCMN: Geriatrics Department, Instituto Nacional de Ciencias médicas y nutrición Salvador Zubirán, Mexico City. The country codes meet the standards of the International Organization for Standardization (ISO). CL: Chile, PE: Peru, CO: Colombia, MX: Mexico, Argentina: AR. N: number of subjects, AD: Alzheimer’s disease, bvFTD: behavioral variant frontotemporal dementia, PD: Parkinson’s disease, MS: Multiple sclerosis, HCs: healthy controls.

### EEG recordings

Both EEG acquisition and processing parameters are summarized in Table 6. Participants were seated in a comfortable chair inside a dimly lit, sound-attenuated, and electromagnetically shielded EEG room and instructed to remain still and awake. Ongoing (resting-state), eyes-closed EEG was recorded for ten minutes using the same amplifier across centers, a 128-channel Biosemi Active-two acquisition system (pin-type active, sintered Ag-AgCl electrodes). The reference electrodes were set to linked mastoids. Furthermore, external electrodes were placed in periocular locations to record blinks and eye movements. Analog filters were set at 0.03 and 100 Hz. The EEG was monitored online for detecting drowsiness, and myogenic and sweat artifacts.

**Table 6:** Equipment and technical parameters for EEG acquisition and processing.

Acquisition	Acquisition system	Biosemi Active II
	Electrode layout	Biosemi 128
	Reference choice	Linked mastoids
	Analog filters (frequency cutoff)	0.03–100 Hz
Processing	Digital filters (frequency cutoff)	0.5 – 40 Hz
	Sampling rate	512 Hz
	Reference choice	Average reference

The EEG was processed offline using an in-house pipeline built upon pre-existing EEGLab functions^85^. Only basic steps were implemented (i.e., re-referencing, filtering, and eliminating bad channels) to allow dataset users to conduct custom analyses. The row data (*.bdf extension) was imported into EEGLab using the BDFimport plugging and processed in the *.set extension (default EEGLab extension). Recordings were re-referenced to the average of all channels (average reference), and band-pass filtered between 0.5 and 40 Hz using a zero-phase shift Butterworth filter of order = 8. Data were down sampled to 512 Hz, and Independent Component Analysis (ICA) was used to correct EEG artifacts induced by blinking and eye movements. Malfunctioning channels were identified using a semiautomatic detection method and replaced using weighted spherical interpolation.

### MRI acquisition

The MRI neuroimages were acquired with 1.5 or 3 Tesla scanners. The list of scanner models and institutions can be found in Table 7. T1-MPRAGE anatomical scans were acquired using a T1-weighted volumetric magnetization-prepared rapid gradient echo sequence. Diffusion and T2-FLAIR images were obtained through T2- and diffusion-weighted images, respectively. The number of slices depended on the acquisition protocol. Resting-state functional MRI completed eyes-open resting state multi-echo BOLD functional scans. Participants were instructed to remain still, keeping their eyes open, with normal breathing to reduce motion artifacts. Resting-state data were recorded using a multi-echo EPI sequence. While individual information has not been incorporated within the main body of the text due to its substantial volume, the details of the acquisition parameters for all subjects are available in the *.json files.

**Table 7:** Equipment used for the MRI acquisition.

MF (T)	Manufacturer	Model
1.5	GE	Signa HDxt
	Siemens	Symphony
		Aera
		Avanto
	Philips	Achieva
		Prodiva CX
		Intera
3.0	Siemens	Skyra
		Verio
	Philips	Achieva
		Ingenia

## Data Records

The neuroimaging data is hosted in the Synapse project “BrainLat-dataset”^28^. This is accompanied by the anonymized demographic information, and both cognitive and functional outcomes. Information is presented in *csv files (plain text, comma-separated values). Additionally, a dictionary containing all column headers from the demographic, cognitive, and neuroimaging csv files has been included in Synapse.

The neuroimaging data is organized according to the Brain Imaging Data Structure (BIDS) specifications^86^ to address the heterogeneity of data organization and follow the FAIR principles of findability, accessibility, and interoperability^87^ while protecting personal information. Initially developed to organize MRI data, the BIDS format has been extended to other neuroimaging modalities, including EEG. Accordingly, EEG data was converted into EEG-BIDS^88^. Conversion of the original files (i.e., e *.dcm for MRI and *.set for EEG) into the BIDS format was made using BIDScoin (for MRI)^89^ and the BIDS EEGLAB plugging^88^ (for EEG). For cases where MRI and EEG data were available from the same participant, the -MRI-BIDS and EEG-BIDS were combined in a single structure. The BIDS structures were validated using BIDS Validator v1.11.0 (https://bids-standard.github.io/bids-validator/). Personal information was removed from the EEG recordings during the EEG-BIDS conversion. The different MRI data were defaced using PyDeface 2.0.0 via Docker v4.12.0 (https://github.com/poldracklab/pydeface).

**Fig. 2:**
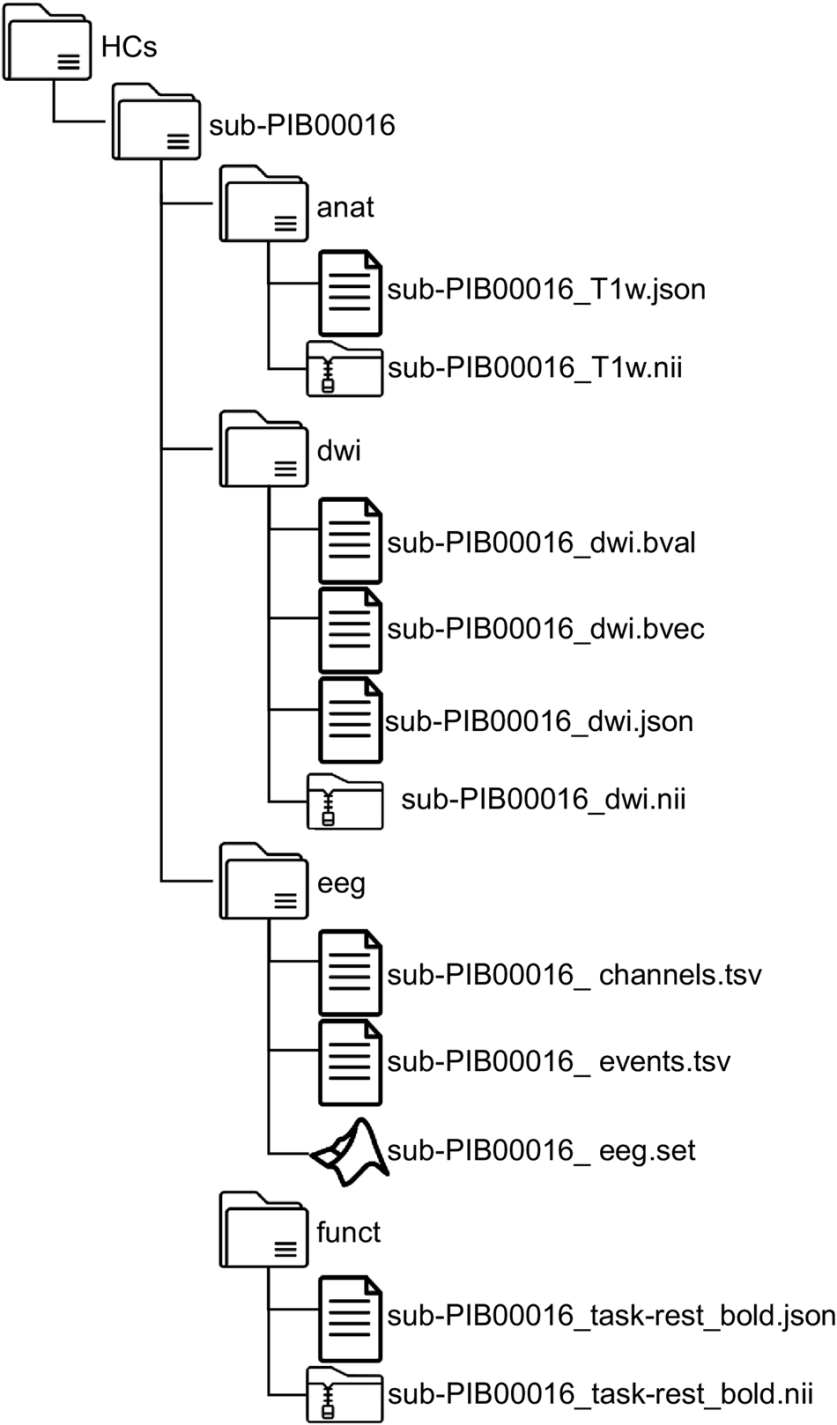
Illustrative diagram of the BrainLat dataset’s directory tree, organized according to the BIDS format. For MRI data, anatomical (anat), diffusion-weighted (dwi), and functional (funct) images are stored in specific files. The same applies to the EEG data.

An example of the directory tree after structuring files according to the BIDS format is presented in Fig. 2. Participants’ data from the same group are stored in the same folder. For a given participant, the data of the different neuroimaging modalities are presented separately, being subfolders named “anat”, “func”, “dwi”, and “eeg”. The name of the files containing the data begins with the “sub-“ index, followed by the letter “P” and two letters referring to the PI responsible for the data acquisition (indicating the recruitment site). The name ends with the number of the subject (e.g., “00035”), followed by a string of characters indicating the neuroimaging modality. In individual folders, the files *.json contain information about the dataset and participants.

## Technical Validation

Quality checks included the implementation of standardized protocols for recruitment and psychophysiological assessment and quality control during the acquisition of neuroimaging data.

### Recruitment

The recruitment comprised the following steps a) selection of HCs controls within the expected range; b) identification of the required control profiles to maintain SD < 2-3 for each match; c) searching for controls to meet the required parameters, such that HCs were matched for age, sex, and education with patients.

### Diagnosis and psychological assessment

Multidisciplinary teams made the diagnosis as part of an ongoing multicentric protocol^38,90^. The cognitive and functional status were assessed following the standard protocols implemented by ReDLat^30^. Evaluators received a clinical certification from board-certified neurologists after completing training and a monitoring process to use standard procedures.

### EEG

Incidences during the EEG acquisition were annotated for further visual inspection. Bad channels were detected using semiautomatic algorithms based on threshold amplitude. Automatic channel rejection and interpolation were implemented. On average, 3.2 ± 1.1 channels were interpolated per recording. Certified experts supervised the quality of the recording.

### MRI

The quality control metrics for the T1w and functional BOLD MRI scans were computed by the MRIQC package^91^, which outputs several quality control metrics of different aspects of the data. These quality control metrics are stored in group_T1w.tsv and group_bold.tsv in the derivatives/mriqc folder.

## Data Availability

This dataset^28^ only comprised raw data. No codes were generated for the creation of the repository. Data processing and generation of BIDS files were done with freely available software.
