# Author Correction: The BrainLat project, a multimodal neuroimaging dataset of neurodegeneration from underrepresented backgrounds

**DOI:** 10.1038/s41597-023-02870-0

**Published:** 2024-01-02

**Authors:** Pavel Prado, Vicente Medel, Raul Gonzalez-Gomez, Agustín Sainz-Ballesteros, Victor Vidal, Hernando Santamaría-García, Sebastian Moguilner, Jhony Mejia, Andrea Slachevsky, Maria Isabel Behrens, David Aguillon, Francisco Lopera, Mario A. Parra, Diana Matallana, Marcelo Adrián Maito, Adolfo M. Garcia, Nilton Custodio, Alberto Ávila Funes, Stefanie Piña-Escudero, Agustina Birba, Sol Fittipaldi, Agustina Legaz, Agustín Ibañez

**Affiliations:** 1 Latin American Brain Health Institute (BrainLat), Universidad Adolfo Ibáñez, Santiago, Chile; 2 Escuela de Fonoaudiología, Facultad de Odontología y Ciencias de la Rehabilitación, Universidad San Sebastián, Santiago, Chile; 3 PhD Neuroscience Program, Physiology and Psychiatry Departments, Pontificia Universidad Javeriana, Bogotá, Colombia; 4 Memory and Cognition Center Intellectus, Hospital Universitario San Ignacio, Bogotá, Colombia; 5 Global Brain Health Institute, University of California San Francisco, San Francisco, USA; 6 Global Brain Health Institute, Trinity College Dublin, Dublin, Ireland; 7 Cognitive Neuroscience Center (CNC), Universidad de San Andrés & CONICET, Buenos Aires, Argentina; 8 Department of Neurology, Massachusetts General Hospital and Harvard Medical School, Boston, MA USA; 9 Departamento de Ingeniería Biomédica, Universidad de Los Andes, Bogotá, Colombia; 10 Memory and Aging Clinic, University of California San Francisco, San Francisco, USA; 11 Neuropsychology and Clinical Neuroscience Laboratory (LANNEC), Physiopathology Department - Institute of Biomedical Sciences (ICBM), Neurocience and East Neuroscience Departments, Faculty of Medicine, University of Chile, Santiago de Chile, Chile; 12 Geroscience Center for Brain Health and Metabolism, (GERO), Santiago de Chile, Chile; 13 Memory and Neuropsychiatric Center (CMYN), Memory Unit – Neurology Department, Hospital del Salvador and Faculty of Medicine, University of Chile, Santiago de Chile, Chile; 14 Servicio de Neurología, Departamento de Medicina, Clínica Alemana-Universidad del Desarrollo, Santiago de Chile, Chile; 15 Centro de Investigación Clínica Avanzada (CICA), Facultad de Medicina-Hospital Clínico, Universidad de Chile, Independencia, Santiago, 8380453 Chile; 16 Departamento de Neurología y Neurocirugía, Hospital Clínico Universidad de Chile, Independencia, Santiago, 8380430 Chile; 17 Departamento de Neurociencia, Facultad de Medicina, Universidad de Chile, Independencia, Santiago, 8380453 Chile; 18 Departamento de Neurología y Psiquiatría, Clínica Alemana-Universidad del Desarrollo, Santiago, 8370065 Chile; 19 Grupo de Neurociencias de Antioquia de la Universidad de Antioquia, Medellín, Colombia; 20 School of Psychological Sciences and Health, University of Strathclyde, Glasgow, United Kingdom; 21 Mental Health Department, Hospital Universitario Fundación Santa Fe de Bogotá, Memory Clinic, Bogotá, Colombia; 22 Departamento de Lingüística y Literatura, Facultad de Humanidades, Universidad de Santiago de Chile, Santiago, Chile; 23 Unit Cognitive Impairment and Dementia Prevention, Peruvian Institute of Neurosciences, Lima, Peru; 24 Geriatrics Department, Instituto Nacional de Ciencias Médicas y Nutrición Salvador Zubirán, Mexico City, Mexico; 25 Instituto Universitario de Neurociencia, Universidad de La Laguna, Tenerife, Spain; 26 Facultad de Psicología, Universidad de La Laguna, Tenerife, Spain

**Keywords:** Neurodegeneration, Biomarkers, Neurodegeneration, Biomarkers

Correction to: *Scientific Data* 10.1038/s41597-023-02806-8, published online 09 December 2023

In this article the author name Maria Isabel Behrens was incorrectly written as Maria Isabel Beherens. The original article has been corrected.

